# Boosting Health Benefits in Vegetables: A Novel Ultraviolet B (UVB) Device for Rapid At-Home Enhancement of Phytochemicals and Bioactivity

**DOI:** 10.3390/foods13203311

**Published:** 2024-10-18

**Authors:** Alejandro Gastélum-Estrada, Edwin E. Reza-Zaldivar, Daniel A. Jacobo-Velázquez

**Affiliations:** Tecnológico de Monterrey, Escuela de Ingeniería y Ciencias, Campus Guadalajara, Av. General Ramón Corona 2514, Zapopan 45201, Jalisco, Mexico; a01229318@tec.mx (A.G.-E.); edwin.reza@tec.mx (E.E.R.-Z.)

**Keywords:** UVB radiation, nutraceutical enhancement, phytochemical content, gene expression, postharvest abiotic stress, anti-obesogenic

## Abstract

The consumption of vegetables is essential for reducing the risk of noncommunicable diseases, yet global intake falls short of recommended levels. Enhancing the nutraceutical content of vegetables through postharvest abiotic stress, such as ultraviolet B (UVB) radiation, offers a promising solution to increase health benefits. This study developed a user-friendly, at-home UVB device designed to increase the phytochemical content in common vegetables like carrots, lettuce, and broccoli. The device applies UVB radiation (305–315 nm) to fresh-cut vegetables, optimizing exposure time and intensity to maximize nutraceutical enrichment. The results demonstrated that UVB exposure increased the phenolic content by 44% in carrots, 58% in broccoli, and 10% in lettuce, with chlorogenic acid levels rising by 367% in lettuce, 547% in broccoli, and 43% in carrots after 48 h of storage. UVB treatment also enhanced antioxidant activity by up to 41% in broccoli and anti-inflammatory potential by 22% in carrots. In terms of gene expression, UVB treatment upregulated UCP-1 expression by 555% in carrots, enhanced thermogenesis, and increased SIRT-1 and ATGL expression by over 200%, promoting lipid metabolism. This process provides a convenient and efficient method for consumers to boost the health benefits of their vegetables. The study concludes that UVB-induced abiotic stress is an effective strategy to improve vegetable nutritional quality, offering a novel approach to increasing bioactive compound intake and aiding in the prevention of diet-related diseases.

## 1. Introduction

Vegetable consumption has been proposed as a tool for reducing the risk of noncommunicable diseases such as cardiovascular diseases, cancers, and type 2 diabetes, among others, due to their rich content of vitamins, minerals, fiber, and phytochemicals [1].

Phytochemicals, including phenolic compounds, glucosinolates, and carotenoids, are present in vegetables and have been associated with antioxidant, anti-inflammatory, and anti-obesogenic activity. They are a main factor in vegetable consumption’s effect against the mentioned noncommunicable diseases [2,3,4].

Among the most consumed vegetables worldwide are carrots (*Daucus carota* L.), lettuce (*Lactuca sativa* L. var. *longifolia*), and broccoli (*Brassica oleracea* var. *Italica*), with a production of 42, 27, and 26 million metric tonnes, respectively, in 2022 [5]. These vegetables have been widely studied due to their rich phytochemical composition with high nutraceutical potential. Carrot consumption is related to high dietary fiber, carotenoids, phenolics, and vitamins, especially provitamin A [6,7]. Broccoli is rich in glucosinolates that, when hydrolyzed, lead to an isothiocyanate, potent anticarcinogenic compounds that induce phase II enzymes in humans responsible for eliminating reactive oxygen species (ROS) [8]. Lettuce, on the other hand, is a low-calorie vegetable rich in fiber, vitamins, and bioactive compounds such as phenolic acids [4].

Despite the bioactivity potential described above, vegetable consumption is insufficient to obtain most of the mentioned health benefits. Although the vegetable consumption recommended by the World Health Organization (WHO) is 400 g/day, the worldwide mean consumption is only 186 g/day and, in regions such as Central America, is as low as 56 g/day; these values do not meet the recommended intake [9].

Postharvest abiotic stress has been proposed as a tool for increasing phytochemical content in raw fresh vegetables [10]. Still, several constraints have been found for their implementation in mass, given the need for incubation at controlled temperatures [11] or controlled atmospheric conditions [12]. These abiotic stresses can potentially increase phytochemical concentrations up to 5–10 times [13,14], achieving a higher health benefit without increasing the amount of vegetable consumption. These processes do not affect the physical properties of the vegetables and can be used as ingredients for other processes [15,16].

Among the reported effective postharvest abiotic stresses are wounding and ultraviolet radiation [17]. While the application of wounding stress requires a subsequent incubation of 24–48 h for phytochemical elicitation [12], ultraviolet (UV) radiation has demonstrated the potential to induce nutraceutical enrichment in an immediate timelapse with no incubation [18,19,20,21]. The difference between wounding and UVB-induced stress obeys the different pathways and defense mechanisms that are activated in vegetable tissue [22,23,24]. Ultraviolet B (UVB, 290–320 nm) radiation has demonstrated superior results compared to ultraviolet A (320–400 nm) and ultraviolet C (220–290 nm), owing to its unique physical properties and the way plants respond to it (for more detailed information, see [17,25]). These responses include the activation of the UV Resistance Locus 8 photoreceptor [26] and the stimulation of key pathways such as the phenylpropanoid, shikimate, and pentose pathways, which are involved in the biosynthesis of phenolic compounds [23,27].

Given the promising results of UVB radiation in enhancing phytochemical content, translating these findings into practical applications for consumers becomes a crucial next step. Developing user-friendly devices that facilitate the application of these treatments at home could greatly enhance the accessibility of nutraceutical benefits. Through the development of a household appliance capable of applying UV stress to vegetables, consumers can increase the nutraceutical content of their produce quickly and conveniently. This would allow individuals to gain maximum health benefits from their vegetables with minimal effort, encouraging more widespread adoption of these strategies.

Based on the previous concept, and recognizing the emerging potential of postharvest exposure to ultraviolet B (UVB) radiation as a strategy to enhance the health benefits of vegetables, this study has several key objectives. First, it aims to develop a device for the domestic application of UVB-induced abiotic stress on three commonly consumed vegetables—carrot, lettuce, and broccoli—for immediate consumption. Additionally, it seeks to optimize the processing conditions for each vegetable and evaluate the effectiveness of this treatment in increasing the phytochemical content. Lastly, the study will assess the impact of this process on the storage of the vegetables, as well as their antioxidant, anti-inflammatory, and anti-obesogenic potential.

## 2. Materials and Methods

### 2.1. Reagents and Plant Material

Carrots, lettuce, and broccoli were purchased from a local supermarket (Walmart, Zapopan, Jalisco, Mexico). HPLC-grade water was obtained from a Milli Q Ultrapure water system (Merck Millipore, Billerica, MA, USA). Solvents, including methanol, methyl tert-butyl ether (MTBE), acetonitrile, acetone, and ethanol (all HPLC and reagent grades), were sourced from CTR Scientific (Zapopan, Jalisco, Mexico). Reagents for colorimetric and chromatography assays, such as phosphoric acid, Sephadex A25, and sodium acetate, were also purchased from CTR Scientific. Likewise, chemical standards, including, chlorogenic acid, β-carotene, desulfoglucoraphanin, sinigrin, and quercetin, were obtained from the same supplier.

Reagents for in vitro assays, including Dulbecco’s modified Eagle medium (DMEM), DMEM high glucose, fetal bovine serum (FBS), streptomycin, newborn calf serum, (3-(4,5-dimethylthiazol-2-yl)-5-(3-carboxymethoxyphenyl)-2-(4-sulfophenyl)-2H-tetrazolium) (MTS), lipopolysaccharides (LPS), Griess Reagent System (Promega; Madison, WI, USA), dichlorodihydrofluorescein diacetate (DCFH-DA), AAPH (2,2′-azobis(2-amidinopropane) dihydrochloride), Hank’s Balanced Salt Solution, isobutylmethylxanthine, dexamethasone, insulin, Oil Red O (ORO), isopropanol, paraformaldehyde (PFA), and phosphate-buffered saline (PBS) were also obtained from CTR Scientific (Zapopan, Jalisco, Mexico). A free glycerol kit was obtained from Abcam (Waltham, MA, USA), RNeasy Mini Kit from QIAGEN (Germantown, MD, USA), triglycerides from Merck-Sigma (Burlington, MA, USA), and a Superscript IV Kit and PowerUp SYBR-Green Kit from ThermoFisher (Waltham, MA, USA).

### 2.2. UVB Treatment Device Design and Construction

**Figure 1:**
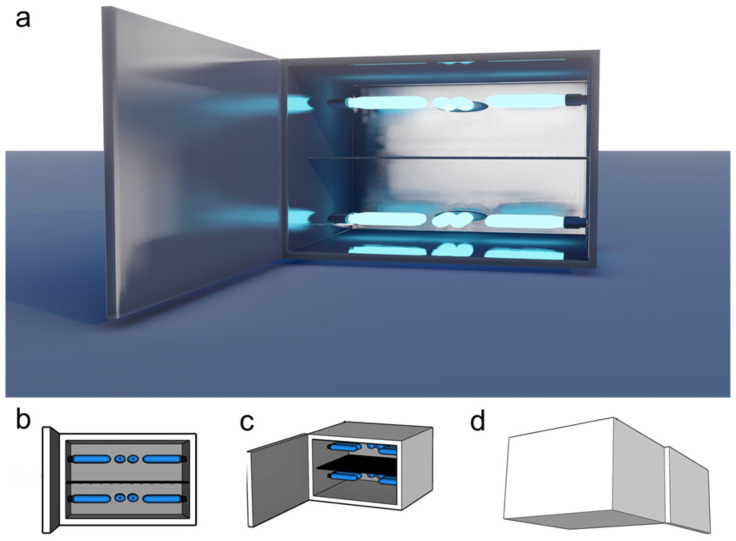
Prototype for at-home UVB vegetable treatment. (**a**) Rendered; (**b**) schematic front; (**c**) front isometric; (**d**) rear isometric views of the proposed prototype device. This device was used for different treatment conditions and adjusted for increased efficiency.

A prototype for at-home UVB treatments was developed and designed for convenient desktop use. The device consisted of a 43 cm × 28 cm × 36 cm chamber equipped with a door for inserting and removing vegetable material. The inner surface was lined with aluminum folds to reflect UVB light. Inside the chamber, a tray made of an 18 × 16 plastic mesh grid was positioned 14 cm from the bottom, at the midpoint of the chamber. This mesh size allowed UVB radiation to pass through while securely holding even small pieces of vegetables (Figure 1).

The device was equipped with six commercial narrowband UVB lamps (PL-S 9W/01/2P Phillips, Pila, Poland). These lamps had dimensions of 12.9 cm × 14.4 cm × 2.8 cm, a power output of 8.6 W, a current of 0.17 A, and a weight of 32 g. The light wavelength range for this lamp model is 305–315 nm, with a peak at 311 nm. Three lamps were installed above and below the tray, positioned 11 cm from the tray surface. 

UVB radiation over the tray was measured using a UVB detector (Model 6.2 UVB, Solarmeter, Glenside, PA, USA) with a 280–320 nm spectral response. Different lamp configurations were tested to achieve higher and more homogeneous UVB exposure. The final lamp arrangement showed a maximum intensity of 12 W/m^2^ for each side (top and bottom) of the tray, with each lamp providing a 2 W/m^2^ intensity on average. This method and device for the rapid increase of nutraceuticals in vegetables has been submitted for patent (MX/a/2024/010935) and industrial design (MX/f/2024/002692) applications to the Mexican Institute of Industrial Property (IMPI, by its acronym in Spanish).

### 2.3. Fresh-Cut Processing and Storage Conditions

The carrots, lettuce, and broccoli were sanitized with sodium hypochlorite (200 ppm, pH 6.5) for 5 min, and excess water was removed before processing with a food processor (FP4200B, Black + Decker, Middleton, WI, USA). The carrots were shredded, while the lettuce and broccoli were chopped. This method was chosen based on each vegetable’s characteristics and common consumption forms. The vegetables were cut to increase the contact surface with the UVB light and facilitate a uniform distribution of UVB exposure, as described in the next section.

For the optimization of UVB treatment conditions (see Section 2.4), a total of 6.5 kg of each vegetable was processed as described and pooled to ensure homogeneity across the entire sample. A 500 g portion was taken as a control and immediately frozen at −80 °C, while another portion of the control was stored at 15 °C for 48 h. The UVB treatments were then applied as outlined in Section 2.3, using 100–120 g per treatment. From each treatment, three independent 5 g samples were randomly selected and frozen for the subsequent extraction of phenolic compounds. The optimization process was based on the quantification of phenolic compounds immediately after UVB treatment and following storage at 15 °C for 48 h.

After determining the optimal UVB conditions for increasing phenolic compounds, the experiment was replicated under these conditions using 2 kg per vegetable. As described in the optimization process, 500 g of tissue was immediately frozen at −80 °C after cutting to serve as the control. Three independent runs were performed under the defined optimal conditions, with 100–120 g used per run. In this new run, additional phytochemicals were quantified alongside phenolics, including carotenoids, and glucosinolates (for broccoli). Furthermore, the effect of the treated vegetables under optimal UVB conditions was evaluated for their antioxidant, anti-inflammatory, and anti-obesogenic capacities in vitro.

### 2.4. Optimization of UVB Radiation Conditions

An experimental design was conducted to optimize UVB intensity exposure. For each vegetable, four different UVB intensities (0, 4, 8, and 12 W/m^2^) and four exposure times (0, 10, 20, and 30 min) were tested. Control conditions (0 min, 0 W/m^2^) were used for the comparison of the effect of different treatments; 0 W/m^2^ for 10, 20, and 30 min conditions were used as a positive control for discarding effects by the short storage into the device in phenolic synthesis.

The cut vegetables were arranged in a monolayer (<0.5 cm thick) on the device tray, with 100–120 g used per treatment. The effect of UVB radiation was evaluated both immediately after the treatment and after storage at 15 °C for 48 h. For each treatment, three independent 5 g samples were randomly collected from the processed tissue, both immediately after UVB treatment and after storage. The samples were frozen at −80 °C for further phenolic compound analysis.

The quantification of phenolic compounds was selected as the evaluation criterion to assess the potential of UVB treatment in enhancing nutraceutical content, due to their role as stress-response metabolites in the selected vegetables. Total phenolic content (TPC) was determined as outlined in Section 2.5.1 and plotted on a surface graph for each vegetable. TPC was modeled using MATLAB R2022b (MathWorks, Natick, MA, USA) in a third-order polynomial regression model with interaction terms, as indicated in Equation (1), where $X_{1}$ represents the independent variable time (min) and $X_{2}$ intensity of UVB radiation (W/m^2^); $\beta_{0}$ for the intercept; $\beta_{1}$ and $\beta_{2}$ as coefficients for linear terms; $\beta_{3}$ and $\beta_{4}$ as coefficients for quadratic terms; $\beta_{5}$ for the linear interaction between $X_{1}$ and $X_{2}$; $\beta_{6}$ and $\beta_{7}$ as coefficients for cubic terms; $\beta_{8}$ and $\beta_{9}$ for quadratic–cubic and cubic–quadratic interactions; and $\beta_{10}$ for the quadratic–quadratic interaction term.

(1) $$TPC=\beta_{0}+\beta_{1}X_{1}+\beta_{2}X_{2}+\beta_{3}X_{1}^{2}+\beta_{4}X_{2}^{2}+\beta_{5}X_{1}X_{2}+\beta_{6}X_{1}^{3}+\beta_{7}X_{2}^{3}+\beta_{8}X_{1}^{2}X_{2}+\beta_{9}{X_{1}X}_{2}^{2}+\beta_{10}X_{1}^{2}X_{2}^{2}$$

The best-considered conditions were selected for further phytochemical and biological activity assays. UVB processing was repeated under these optimal conditions, as described in Section 2.3, in three independent runs. Samples (5 g) for phenolic determination were freshly taken and immediately frozen at −80 °C, while the remaining tissue was freeze-dried for additional phytochemical analyses (carotenoids and glucosinolates) and in vitro assays (see Section 2.5 and Section 2.6, respectively).

### 2.5. Extraction, Identification, and Quantification of Phytochemicals

#### 2.5.1. Phenolics

Phenolic compounds were determined according to Becerra-Moreno et al. [28]. Briefly, vegetable tissue (5 g) was homogenized with methanol (20 mL) using a tissuemizer (IKA, Ultra Turrax, Staufen, Germany) and then centrifuged (3134× *g*, 4 °C, 30 min).

The supernatant was recovered and filtered using a nylon syringe filter (0.22 µm) and injected (10 µL) in an Ultra-High Pressure Liquid Chromatograph–Photo Diode Array (UHPLC-PDA) Acquity Arc system (Waters, Milford, MA, USA). The compounds were separated at 40 °C on a Waters Cortecs reverse-phase C18 (4.6 mm × 150 mm, 2.7 µm pore size) column. The mobile phases were (A) Mili-Q water adjusted to pH 2.4 using orthophosphoric acid and (B) methanol. The gradient elution was 0/90, 13.2/65, 15.6/2, and 19.2/90 (min, % phase A) at 1 mL/min flow. Chlorogenic acid (CHA) and total phenolics were determined (mg/kg DW) as the individual peak and the sum of all identified compounds, respectively, and quantified as CHA equivalents using a calibration curve (2–200 ppm).

#### 2.5.2. Carotenoids

Carotenoids were extracted and analyzed as indicated by Santana-Gálvez et al. [13] with slight modifications. Extractions were performed under dark conditions at room temperature. Briefly, 0.2 g of freeze-dried sample was weighed and homogenized with 20 mL of acetone for 30 s using a tissuemizer. Homogenates were filtered through a Whatman No. 1 paper filter under vacuum. The process was repeated using 10 mL of extraction solution 3 times to ensure complete extraction. The extracts were collected on a 50 mL volumetric flask, and the volume was completed with acetone solution. The extracts were filtered using a nylon syringe filter (0.22 µm) and immediately injected (20 µL) in a UHPLC-PDA system.

The compounds were separated on the same Waters Cortecs C18 column used for the phenolics analysis. The samples and column temperature were 4 and 30 °C, respectively. The mobile phase was methanol, MTBE, and water in an isocratic system of 50, 45, and 5%, respectively. The elution time was 15 min at a flow rate of 1 mL/min. Chromatograms were detected at 450 nm, and the quantification of β-carotene and total carotenoids was performed using a β-carotene standard curve (1–100 ppm).

#### 2.5.3. Glucosinolates

Glucosinolates were only quantified in the broccoli, as carrots and lettuce are not significant sources of these compounds. In the case of broccoli, glucosinolates were extracted, desulfated, and quantified as desulfoglucosinolates following the method described by Villarreal-García et al. [29]. For extraction, 0.3 g of freeze-dried broccoli was added to 8 mL of 70% ethanol/water (*v*/*v*) solution at 70 °C and vortexed. The mixture was cooled and centrifuged (3134× *g*, 15 min, 4 °C), and the supernatant recovered. Glucosinolates were desulfated using disposable propylene columns previously prepared with Sephadex A-25 in sodium acetate (8.7% *w*/*v*). Sample supernatant (3 mL) was added to the prepared column with sinigrin as internal standard (50 µL, 3 mM) and washed twice with 500 µL of water and sodium acetate. Sulphatase (75 µL) was added and incubated for 14 h at room temperature. Desulfoglucosinolates were eluted after incubation using 1.25 mL of water.

A volume of 10 µL was injected into the UHPLC-PDA Acquity Arc system and separated using a Waters Cortecs C18 column. The mobile phases consisted of water (A) and acetonitrile (B) using gradients 0/100, 17/20, 18/0, and 19.2/0 (min, % phase A) at a constant flow of 1.5 mL/min. Total glucosinolates were quantified as desulfo-glucorpahanin equivalents using a standard calibration curve (1–125 ppm) at 227 nm.

### 2.6. Cell Culture

Anti-inflammatory potential, cellular antioxidant activity, and lipid metabolism evaluation assays were carried out in vitro for the selected condition of UVB treatments using human colorectal adenocarcinoma (Caco-2), mouse macrophage (Raw 264.7), and mouse embryonic fibroblast (3T3-L1), respectively. The cells were purchased from the American Type Culture Collection (ATCC, Manassas, VA, USA). Caco-2 and Raw 264.7 cells were maintained in DMEM/F12 supplemented with 10% FBS and 1% antibiotic (10,000 units penicillin and 10 mg streptomycin/mL), while the 3T3-L1 cells were maintained in DMEM high glucose supplemented with 10% calf serum and 1% antibiotic at 37 °C and 5% CO_2_.

#### 2.6.1. Extract Preparation

Methanolic extracts for each vegetable were performed as mentioned in Section 2.5.1. The solvent was evaporated using a rotary concentrator (Vacufuge, Eppendorf, Hamburg, Germany) at 45 °C and redissolved in DMSO in a 10:1 ratio with respect to the original extract.

#### 2.6.2. MTS Assay

The cytotoxicity of each vegetable DMSO-resuspended extract was evaluated in all cell lines using the MTS reagent. Upon reaching confluence, the cells were plated onto 96-well plates at densities of 2 × 10^4^ to 3 × 10^4^ cells per well. After 24 h, several treatments including 41.7, 62.5, 100, and 125 µg/mL of each vegetable extract were applied to each cell culture and incubated again for 24 h. The medium was removed, replaced with a 10% MTS (5 mg/mL) solution, and incubated for 2 h. Absorbance was measured at 490 nm using a microplate reader (Varioskan Lux, ThermoFisher, Vantaa, Finland), and the cell viability was compared to the non-treated control.

#### 2.6.3. Evaluation of Anti-Inflammatory Potential

The potential anti-inflammatory activity was measured using Raw 264.7 cells stimulated by lipopolysaccharide (LPS) and Griess reaction. Briefly, cells were seeded in a 96-well plated and incubated for 24 h. The cells were incubated for 1 h with a non-toxic dilution of DMSO-resuspended vegetable extract. Then, the treatments were removed, replaced with 2.5 µg/mL LPS, and the samples incubated for 24 h. Nitrite production was measured using the Griess Reagent System (Promega, G2930) according to the manufacturer’s instructions. The anti-inflammatory potential was reported as the nitrite oxide production inhibition of treated cells against a non-treated control.

#### 2.6.4. Cellular Antioxidant Activity (CAA)

The relative quantity of reactive oxygen species (ROS) produced by cells treated with DMSO-resuspended extracts compared to non-treated control was measured using Caco-2 cells. Briefly, 3 × 10^4^ cells were seeded in a black 96-well plate. A solution of DFCH-DA (80 µM) and the non-toxic dilution obtained by MTT assay was added to the plate, incubated for 1 h, and then washed twice using PBS. Then, a 600 µM AAPH solution in Hank’s Balanced Salt Solution was added to the plate. Fluorescence excitation (485 nm) and emission (538 nm) were immediately measured every 5 min for 1 h at 37 °C (13 readings in total). CAA was quantified by examining the percentage of reduction in the fluorescence of treated cells against non-treated control as indicated in Equation (2), where CAA units were reported as mean ± standard error.

(2) $$CAA\;unit=\%\;reduction=1-\frac{AUC\;sample}{AUC\;control}\times 100$$

#### 2.6.5. Evaluation of Antiadipogenic Potential

Lipid accumulation was determined by Oil Red O (ORO) staining after the 3T3-L1 cell differentiation. First, the cells were seeded in a 48-well plate with DMEM supplemented with 10% newborn calf serum. Adipocyte differentiation was induced by DMEM high glucose (DMEM-HG) supplemented with 10% FBS, 0.5 mM isobutyl-methyl-xanthin, 1 µM dexamethasone, and 10 µg/mL of insulin. After three days, the differentiation medium was switched to DMEM-HG supplemented with 10% FBS and 10 µg/mL of insulin. Three days later, the medium was replaced with DMEM-HG supplemented with 10% FBS. Treatments were added during every medium replacement at the non-toxic concentrations defined at the MTS assay. ORO staining was performed on day twelve of differentiation. A working solution was prepared by mixing stock solution (0.5% ORO) with distilled water at a 6:4 ratio, and filtered using a 0.22 µm syringe filter. The cells were washed twice with PBS 0.01 M (pH 7.4) and fixed with PFA 4% for 15 min. The prepared ORO working solution was incubated for 30 min, and the cells were rewashed thrice with PBS. The cells were observed by microscope (OPTIKA IM-3, OPTIKA, Ponteranica, Italy) and documented with an assembled camera (Optikam PRO8 Digital Camera C-P8, OTIKA, Ponteranica, Italy) using the manufacturer’s software (OPTIKA LiteView v2.1.217, OPTIKA, Ponteranica, Italy). The stained lipid droplets were incubated with isopropanol 60% to extract the dye and absorbance was measured at 490 nm. Free lipid accumulation was reported as a percentage of treatments compared to non-treated control absorbance.

#### 2.6.6. Triglyceride Quantification

The triglyceride levels were quantified using a lipase-based assay kit (Abcam, Cambridge, UK). The harvested cells were resuspended in 1 mL of 5% Triton X-100 solution and subjected to a heating cycle at 90 °C for 5 min, followed by cooling to room temperature. This heating and cooling process was repeated twice to ensure thorough cell lysis. The lysate was then centrifuged at 10,000× *g* for 2 min, and the resulting supernatant was carefully transferred to a new tube and diluted tenfold with the assay buffer. For the assay, 50 μL of the diluted supernatant was mixed with 2 μL of a cholesterol esterase/lipase solution and incubated at room temperature for 20 min. Following this, 50 μL of the Triglyceride Reaction Mix was added to the mixture and incubated for 60 min in the dark. Absorbance at 570 nm was then measured to determine the triglyceride concentration in the samples.

#### 2.6.7. Free Glycerol Measurement

Free glycerol levels were quantified post-differentiation using a colorimetric assay kit (Abcam, Cambridge, UK). Briefly, 25 μL of the cell culture supernatant was combined with 100 μL of Free Glycerol Assay Reagent in a 96-well plate and incubated for 15 min at RT. Absorbance was measured at 540 nm. Glycerol concentrations were determined by comparison to a standard curve ranging from 0 to 125 μg/mL and were expressed as a percentage relative to the control.

#### 2.6.8. qPCR

To analyze the lipid-metabolism-related gene expression, total RNA was extracted from fully differentiated 3T3-L1 cells using the RNeasy Mini Kit (QIAGEN), following the manufacturer’s instructions. RNA concentration and purity were assessed using a NanoDrop spectrophotometer (Thermo Scientific, Wilmington, DE, USA). Complementary DNA (cDNA) was synthesized using the Superscript IV Kit according to the manufacturer’s protocol. Gene expression analysis for lipid-metabolism-related genes was conducted using the PowerUp SYBR-Green Kit. The specific primers used for the qPCR analysis of PPARγ, C/EBPα, FAS, UCP-1, PGC-1α, ATGL, and SIRT-1 are listed in Table S1. The PCR conditions included an initial UDG activation at 50 °C for 2 min, followed by denaturation at 95 °C for 2 min. This was succeeded by 40 cycles of denaturation at 95 °C for 15 s and annealing at 60 °C for 30 s. All reactions were performed in triplicate, with β-actin serving as the housekeeping gene. Relative gene expression levels were calculated using the 2^−ΔΔCT^ method.

### 2.7. Statistical Analysis

Three independent experiments were conducted (*n* = 3). Statistical analyses were performed using the mean value of samples and their standard error. Analyses of variance (ANOVAs) were conducted to determine main effects and statistical differences; treatment comparisons were performed using the LSD test (*p* < 0.05). Statistical analyses were conducted using JMP software version 17.0 (SAS Institute Inc., Cary, NC, USA).

## 3. Results and Discussion

### 3.1. UVB Exposure Optimization

**Figure 2:**
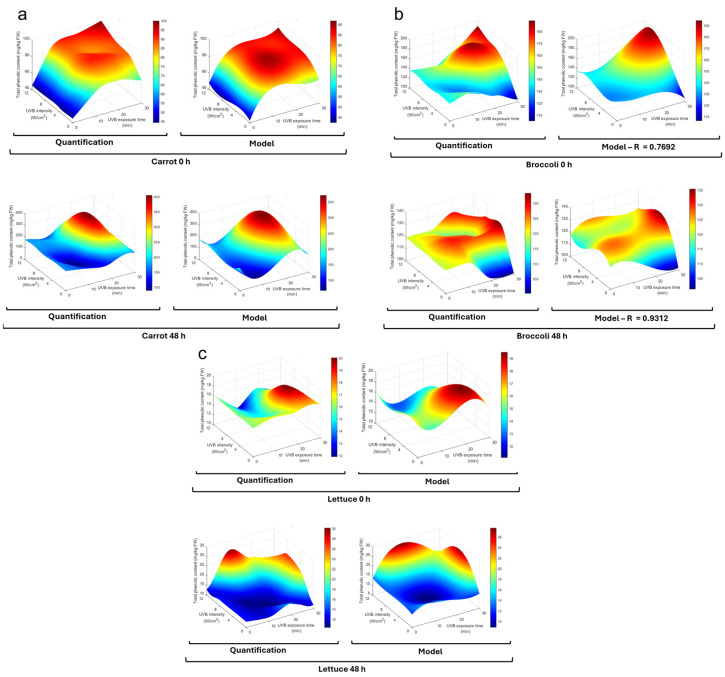
Phenolic content is affected by UVB exposure under different conditions. Total phenolic content in (**a**) carrot, (**b**) broccoli, and (**c**) lettuce subjected to UVB treatments at different exposure times (0–30 min) and intensities (0–12 W/cm^2^), both immediately after exposure (0 h) and after storage at 15 °C (48 h). Color scale is indicated to the side of each plot. HPLC-DAD quantification of treatments and a third-order polynomial regression model are plotted, and model coefficients are shown in Table 1.

UVB radiation exposure significantly affected the total phenolic compound content in carrots, broccoli, and lettuce after exposure and 48 h of storage at 15 °C (Figure 2). The time and intensity affected each vegetable differently, as seen in the proposed model (Table 1). For carrots, the immediate effect was more greatly enhanced by time (*β*_1_) of exposure than intensity (*β*_2_), while the quadratic terms (*β*_3_ and *β*_4_) indicated that the effect of time and intensity was positive at the early stage and then reduced the phenolic content; this was also observed in higher-order interaction terms (*β*_9_ and *β*_10_). The interaction term *β*_5_ indicated a positive but modest interaction between time and intensity. The cubic terms (*β*_6_ and *β*_7_) indicated a slight probability of increase at greater time or intensity levels, which was not observed in the results. After 48 h of incubation, the trends varied, and the baseline was significantly higher than the immediate levels (*β*_0_ = 197.2825). Linear time and intensity negatively affected the phenolic content, but the quadratic terms indicated an increase after an initial decrease. In this model, a strong and positive interaction (*β*_5_) was observed between factors.

It is known that wounding and chopping induce stress responses in plants, leading to the activation of defense mechanisms that can influence secondary metabolite production in different vegetables [30,31]. In carrots, the immediate effect may be triggered by the wounding of carrots and the release of ROS and other molecular signals [22], priming the tissue for enhanced phenolic synthesis upon subsequent UVB stress [32] (Figure 2a). The combination of wounding stress and UVB radiation may have had a synergistic effect, leading to a more pronounced accumulation of phenolics as part of the plant’s defense response [33]. The quadratic terms indicate the existence of an optimal window for UVB treatment. Excessive UVB exposure could lead to photodamage or the activation of catabolic pathways that degrade phenolic compounds [27]. After 48 h of storage, the higher baseline in carrots suggests continued synthesis and accumulation during incubation; this process has been previously reported and attributed to wound-induced pathways [24].

Regarding broccoli (Figure 2b), the immediate effect (0 h) linear terms indicated a negative effect by time and UVB intensity, but the quadratic terms indicated that UVB exposure increased the total phenolic content after an initial decrease. A weak but positive interaction was observed for the interaction terms, with minimal effect by cubic and higher-order interaction terms. As for carrots, the linear effect changed for incubated broccoli; in this case, the linear terms became positive and the quadratic terms were negative, suggesting that the phenolic content started to decrease at high intensities; all interaction and cubic terms were minimal.

For broccoli, the immediate negative linear effect followed by a positive quadratic effect suggests an initial depletion of phenolic compounds, possibly due to UVB oxidative stress. However, this may also be due to chopping mechanical stress, followed by the activation of synthesis pathways [21,34]. The positive linear terms after storage suggest a continued phenolic accumulation by wound-induced signaling and a possible synergistic effect of UVB given the positive linear term of intensity [35].

Finally, for lettuce’s immediate effect (0 h), the time linear term indicated a slight decrease, while intensity provoked a small increase (Figure 2c). In the case of quadratic terms, the time term was small but positive, while the intensity was negative; this indicates that the trend indicated by the linear terms changed after an initial stage and did not significantly impact the phenolic concentration. After incubation (48 h), both time and intensity linear terms were positive and quadratic terms slightly negative. This indicates a positive impact on the phenolic concentration at the first stage, but this became negative at high levels. The linear interaction term was negative, suggesting that factors may slightly diminish each other’s effect. The cubic and higher-order terms were small and had a low impact on phenolic content.

In lettuce, the immediate slight decrease with time and small increase with intensity may reflect a balance between consumption and synthesis induced by UVB and mechanical stress [36,37]. After storage, the positive influence of time and intensity suggests that the combined effect of wounding and UVB exposure stimulated phenolic biosynthesis and its accumulation over time.

Wounding stress has been previously reported, and its influence on phenolic accumulation has been considered [21,22,30,31,34,36]. Nevertheless, the interaction with UVB radiation is observed to be synergistic with the biosynthesis and accumulation of the phenolic compounds in the three studied vegetables.

Given the obtained results, 20 min and 8 W/cm^2^ intensity were the conditions considered for use in the further analysis and evaluations of UVB treatment.

### 3.2. Phytochemical Evaluation of UVB-Treated Vegetables

**Figure 3:**
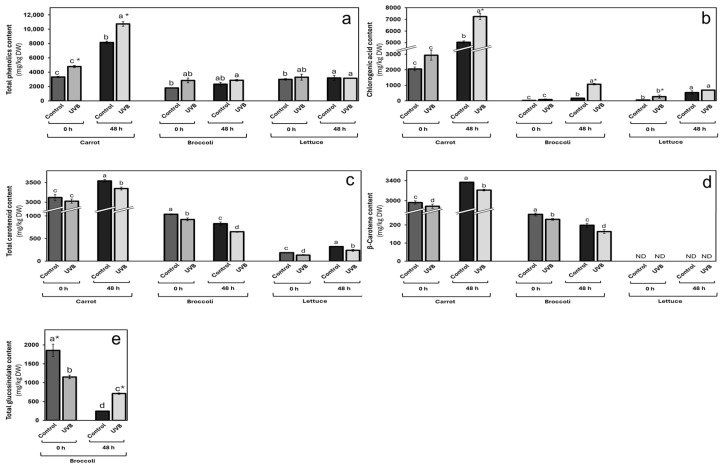
Changes in phytochemical content in carrot, broccoli, and lettuce by UVB exposure. (**a**) Total phenolic; (**b**) chlorogenic acid; (**c**) total carotenoid; (**d**) β-carotene, and (**e**) total glucosinolate content in vegetables exposed (UVB) and non-exposed (control) to UVB for 20 min at 8 W/m^2^. Data represent the mean of 3 repetitions ± the standard error of the mean. Different letters among bars (a–d) indicate a statistical difference between treatments within the same vegetable using the LSD test (*p* < 0.05). Asterisk (*) indicates statistical differences between treatments for the same vegetable at the same evaluated time.

The total phenolic content (Figure 3a) increased for all vegetables immediately after UVB treatment and after 48 h storage (15 °C), except for stored lettuce. In the case of carrots, the increase was 44 and 32% at 0 and 48 h, respectively, against non-UVB-exposed vegetables. Broccoli showed a 58% immediate increase, while the stored sample increased by 23%. For lettuce, the immediate increase was 10%, while no significant increase was observed after storage, as mentioned above.

Chlorogenic acid (Figure 3b) was the most impacted by UVB exposure within the phenolic analysis, showing relevant increases for the three evaluated vegetables at both times (0 and 48 h). For carrots, the increase rates were similar to those for total phenolics, with 43 and 44% immediately after exposure and after storage. For broccoli, the immediate increase was 305% and even higher (547%) after 48 h of storage. Regarding lettuce, the immediate increase was 367% and 30% after storage.

The increase in total phenolics immediately after UVB treatment and after 48 h storage in carrots and broccoli suggests the induction of defense responses due to wounding and UVB exposure. Wounding has been shown to upregulate phenolic biosynthesis genes, increasing the accumulation of compounds like chlorogenic acid [27,38]. The substantial increase in chlorogenic acid observed in all three vegetables suggests that this compound plays a central role in the defense response to combined mechanical and UVB stress.

As mentioned before, the combination of wounding and UVB exposure may have a synergistic effect on phenolic accumulation. In carrots, similar increase rates of total phenolics and chlorogenic acid immediately after exposure and storage indicate sustained metabolic activity in response to stress [17,24]. In broccoli and lettuce, the significant increases in chlorogenic acid after storage suggest that these tissues continue synthesizing phenolics over time, possibly due to prolonged wound-induced signaling being slightly elicited by UVB exposure. UVB exposure for all vegetables affected the total carotenoid content (Figure 3c) by up to 27%. A slight 3 and 6% decrease was observed for carrots at 0 and 48 h after UVB exposure, respectively. Broccoli showed an immediate reduction of 11%, while a 21% decrease was observed after storage. Lettuce was the most affected in terms of carotenoid content, with 27 and 26% at 0 and 48 h after exposure, respectively.

β-carotene was also measured (Figure 3d) as a relevant carotenoid, and, like the total carotenoid content, it was negatively affected by UVB exposure. For carrots, a similar reduction to total content was observed, with an immediate decrease of 3% and 5% after 48 h storage. In broccoli, the reduction was 10 and 18% for 0 and 48 h after UVB exposure, respectively. This compound was not detected in lettuce in any condition. The decrease in total carotenoid content and β-carotene across all vegetables may result from oxidative degradation due to increased ROS generated by wounding and UVB exposure [39]. Carotenoids can act as antioxidants, and their use for ROS neutralization may lead to concentration reduction. The greater reduction in lettuce could be due to its thinner tissue and higher surface area exposed to oxidative stress.

Glucosinolates are known bioactive compounds in broccoli. They were analyzed after UVB exposure (Figure 3e). While a decrease (38%) was observed after treatment, a 190% increase was measured after 48 h of storage.

In broccoli, the initial decrease in glucosinolates after UVB treatment, followed by a significant increase after storage, suggests that wounding may initially lead to glucosinolate hydrolysis due to the release of myrosinase enzymes, while subsequent synthesis during storage replenishes these compounds [40].

### 3.3. Impact of UVB Exposure on the Bioactivity of Vegetables

The current study evaluated how UVB exposure affects the anti-inflammatory, cellular antioxidant, and anti-obesity properties of three common vegetables: carrots, broccoli, and lettuce. The findings provide important insights into the impact of UVB treatment and storage conditions on nitrite production (a key marker of inflammation), cellular antioxidant capacity, and lipid metabolism.

In the first instance, the MTS assays used to establish the optimal working concentrations of each vegetable extract for assessing their cellular antioxidant activity, anti-inflammatory potential, and influence on adipocyte differentiation showed minimal cytotoxicity, with a reduction in cell viability of 20% or less in all three cell lines tested (Figure S1). Based on these observed results, a concentration of 125 µg/mL was chosen for subsequent testing.

#### 3.3.1. Anti-Inflammatory and Antioxidant Properties of UVB-Treated Vegetables

**Figure 4:**
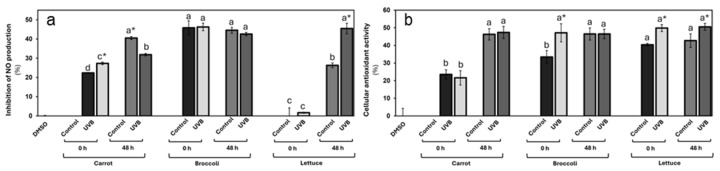
Anti-inflammatory potential (**a**) and cellular antioxidant activity (**b**) for UVB-exposed vegetables at 125 µg/mL concentration. Cellular antioxidant activity and inhibition of nitric oxide as anti-inflammatory potential were evaluated in Caco-2 and Raw 264.7 cells, respectively. Data represent the mean of 3 repetitions ± the standard error of the mean. Different letters among bars (a–d) indicate a statistical difference between treatments within the same vegetable. Asterisk (*) indicates statistical differences between treatments for the same vegetable at the same evaluated time. All statistical differences were defined using the LSD test (*p* < 0.05).

The vegetable extracts’ anti-inflammatory and antioxidant potential is presented in Figure 4. In the NO2 production assay, UVB exposure had a marked effect on the carrot’s anti-inflammatory potential, with a 22% increase compared to the non-treated control (Figure 4a). However, after storage, the anti-inflammatory potential decreased by 21% in contrast with the storage control group, yet remained higher than both the non-treated and the non-stored UVB-treated group. Stored carrots exhibited the highest anti-inflammatory potential. These results agreed with previously reported findings [41]. Interestingly, this pattern suggests that UVB exposure triggers a rapid increase in bioactive compounds capable of enhancing the anti-inflammatory properties of carrots, particularly when used immediately or after a limited storage time.

In contrast, broccoli showed a high anti-inflammatory potential (4% of NO_2_ production inhibition). However, there are minimal changes in anti-inflammatory potential following UVB exposure or storage. The reduction of only 4% after storage suggests that UVB exposure has a limited effect on the anti-inflammatory properties of broccoli, both immediately and after incubation. This could indicate either a lower sensitivity of broccoli’s bioactive compounds to UVB radiation or a more stable phytochemical profile that is less prone to modification by external factors such as light or storage, as demonstrated in the phytochemical characterization.

Lettuce displayed a unique response. Initially, the non-treated lettuce showed negligible anti-inflammatory potential, and after UVB exposition, this activity increased by 2%. However, after the storage, its capacity to reduce nitrite production significantly increased, with the control group demonstrating a 26% reduction. Notably, the UVB-exposed lettuce exhibited an even stronger anti-inflammatory effect, with a 45% reduction in nitrite production. This improvement post-storage suggests that storage and UVB exposure may induce the accumulation of compounds with strong anti-inflammatory properties such as CHA. The observed effect that UVB-treated lettuce consistently outperformed the control highlights the effectiveness of UVB in boosting lettuce’s bioactivity, particularly over time.

On the other hand, the cellular antioxidant activity assays further supported these observances, revealing vegetable-specific responses to UVB exposure (Figure 4b). For carrots, the assay results demonstrate that wounding stress treatment significantly increased the carrot’s antioxidant potential similar to the previously reported [41]. A slight, but not significant, reduction was observed immediately after UVB exposure. After storage, an increase in the antioxidant activity was displayed in both groups (107% on average, contrasting with the non-stored groups), with a slight increase in the antioxidant activity in the UVB-irradiated group, but still with no significant differences. These results imply that carrots may not experience an immediate boost in antioxidant capacity from UVB exposure.

For broccoli, high antioxidant activity was demonstrated among all groups. In the non-storage condition, broccoli exhibited a sharp 41% increase in antioxidant activity immediately following UVB exposure, suggesting that UVB treatment can rapidly enhance its cellular antioxidant properties. However, after storage, the difference between treated and untreated tissue disappeared, although both still maintained higher antioxidant activity than the control at 0 h. This finding suggests that while UVB exposure can induce a short-term increase in antioxidant capacity in broccoli, the effect is not sustained during storage. Nevertheless, the overall improvement compared to the control highlights UVB’s potential to enhance antioxidant properties in the short term.

Lettuce displayed a consistently elevated antioxidant activity in UVB-exposed tissues at both 0 and 48 h post-exposure. Increases of 24% and 18%, respectively, suggest that UVB treatment effectively enhances the antioxidant potential of lettuce and that this effect persists over time. These sustained increases in antioxidant activity could be attributed to the activation or accumulation of bioactive compounds that are either resistant to degradation during storage or that continue to be produced over time.

These findings align with previous studies suggesting that UVB exposure can enhance the bioactivity of certain vegetable tissues [21,42]. UVB treatment offers a promising method for enhancing the anti-inflammatory and antioxidant activity by triggering stress responses in plants and activating defense pathways that increase the biosynthesis of phenolic compounds like CHA and other compounds [43], which are known to play a significant role in cellular and molecular processes related to oxidative stress and inflammation [44,45,46]. For example, in this study, the high level of CHA observed in all three vegetables (Figure 3) can be related to improved anti-inflammatory and cellular antioxidant activity due to its capacity to modulate key signaling pathways related to the activation of the nuclear factor kappa-light-chain-enhancer of activated B cells (NF-κB) pathway, which is known to drive inflammation by promoting the expression of pro-inflammatory cytokines such as TNF-α, IL-6, and IL-1β [47], as well as its potent free-radical-scavenging properties [45].

These results suggest that UVB treatment can be an effective method for enhancing the health-promoting properties of certain vegetables. The variation in responses between vegetables emphasizes the need for optimizing UVB exposure strategies depending on the specific vegetable and desired bioactive outcome.

#### 3.3.2. Effects of UVB-Treated Vegetables on Lipid Accumulation and Metabolism

Using fully differentiated 3T3-L1-like adipocytes, the effects of UVB-exposed vegetable extracts on adipogenesis, lipid metabolism, and the expression of lipid-related genes were assessed.

**Figure 5:**
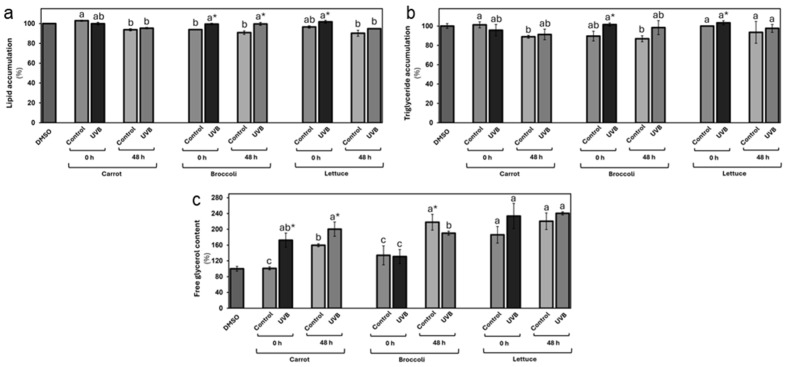
In vitro evaluation of lipid accumulation and distribution as affected by carrot, broccoli, and lettuce treated with UVB extracts at 125 µg/mL concentration. Lipid accumulation (**a**), triglyceride accumulation (**b**), and free glycerol content (**c**) were evaluated in differentiated 3T3-L1 cells. Data represent the mean of 3 repetitions ± the standard error of the mean. Different letters among bars (a–d) indicate a statistical difference between treatments within the same vegetable. Asterisk (*) indicates statistical differences between treatments for the same vegetable at the same evaluated time. All statistical differences were defined using the LSD test (*p* < 0.05).

In terms of lipid accumulation (Figure 5), the results of the ORO staining demonstrated that only stored carrot extracts displayed a slight lipid accumulation reduction of 6%, contrasting with the vehicle group (DMSO). The UVB exposure did not significantly affect lipid levels in carrot extracts at either 0 or 48 h post-treatment when compared with the respective control group. In the broccoli, both controls (0 and 48 h) displayed a reduction in the lipid levels of 6 and 9%, respectively. Interestingly, there was a slight increase in lipid accumulation (6% and 10% at 0 and 48 h, respectively) following UVB treatment. Similarly, only the stored lettuce extract displayed a 10% reduction in lipid accumulation compared with the vehicle group. There were no significant changes in lipid accumulation between UVB-exposed and non-exposed tissues at either time point.

The triglyceride accumulation followed similar patterns to the total lipid accumulation (Figure 5b). Only the carrot extracts from stored samples displayed a significant triglyceride level reduction (11 and 9%, respectively) contrasting with the vehicle group. There were non-detectable significant changes following the UVB treatment. The broccoli extract controls at 0 and 48 h displayed a reduction in triglyceride levels of 10 and 13%, respectively. A substantial 13% increase in triglyceride content following UVB treatment was observed at 0 and 48 h. Lettuce also displayed a small increase (3%) at 0 h in terms of triglyceride accumulation with UVB exposure. No significant differences in triglyceride levels were detected for non-treated controls at 0 and 48 or for the UVB-exposed samples after storage.

Surprisingly, the free glycerol evaluation revealed increased free glycerol levels (Figure 5c) after the vegetable extract treatment. This effect was more markedly affected by UVB exposure, particularly in carrots, where a significant increase of 71% and 26% was observed at 0 and 48 h, respectively. This suggests that UVB treatment may enhance lipolysis in the carrot group, leading to greater glycerol release. In the broccoli treatment, there was no change in free glycerol content at 0 h, but a 13% reduction was observed after 48 h, contrasting with the control at 48 h, indicating a possible decline in lipolytic activity during storage. The lettuce group displayed an immediate (0 h) 26% increase in free glycerol following UVB exposure, with a smaller 9% increase after storage.

The modulation of lipid metabolism, particularly fat deposition and its mobilization, has drawn significant attention as a potential therapeutic approach for obesity management [48]. This study’s findings support previous studies that report how vegetable abiotic stresses can influence the biosynthesis of phytochemicals that can modulate lipid accumulation and lipolytic activity in mammal cells [41,46], with implications for understanding the role of dietary components in lipid metabolism [49,50,51]. While the results indicated that UVB treatment did not significantly alter the vegetable extract effect in terms of total lipid or triglyceride levels, it did highlight notable changes in free glycerol content, particularly after the carrot and lettuce treatments. These shifts in glycerol levels suggest that UVB-exposed vegetable extracts may stimulate lipolysis, especially in those vegetables that contain phenolic compounds, such as CHA, known to modulate lipid metabolism [52].

Interestingly, phenolic compounds, including CHA, have been shown to exert both inhibitory and stimulatory effects on adipogenesis and lipid metabolism depending on their concentration and duration of exposure, among other factors [46,53]. This is based on the phenomenon known as hormesis, which is defined as a biphasic response to phytochemicals in relation to physiological functions [54]. For example, CHA at low to moderate concentrations is generally regarded as anti-adipogenic because it can reduce the lipid droplet formation and lipid accumulation in adipocytes by downregulating key transcription factors such as peroxisome proliferator-activated receptor gamma (PPAR-γ), RXRγ, CCAAT/enhancer-binding proteins (C/EBPs), and adipogenic genes such as FABP4 and FASN [55]. Moreover, CHA can induce the activation of key players that promote lipolysis such as ATGL and HSL, enhancing the breakdown of stored triglycerides and preventing the accumulation of new lipid stores [46]. However, the effects of CHA and other phenolics can become paradoxical at higher concentrations, exerting pro-adipogenic effects including the upregulation of PPAR-γ and C/EBPs, likely by acting as partial PPAR-γ agonists without modifying the impact of CHA in adipocyte lipolysis [56]. This situation may explain why there is only a reduction of lipid accumulation in the groups treated with vegetable extract with non-enriched phenolic compounds and why the potential lipolytic activity is even higher in some UVB-exposed vegetable extracts.

#### 3.3.3. Effect of Vegetable UVB Exposure on the Expression of Genes Related to Lipid Metabolism

**Figure 6:**
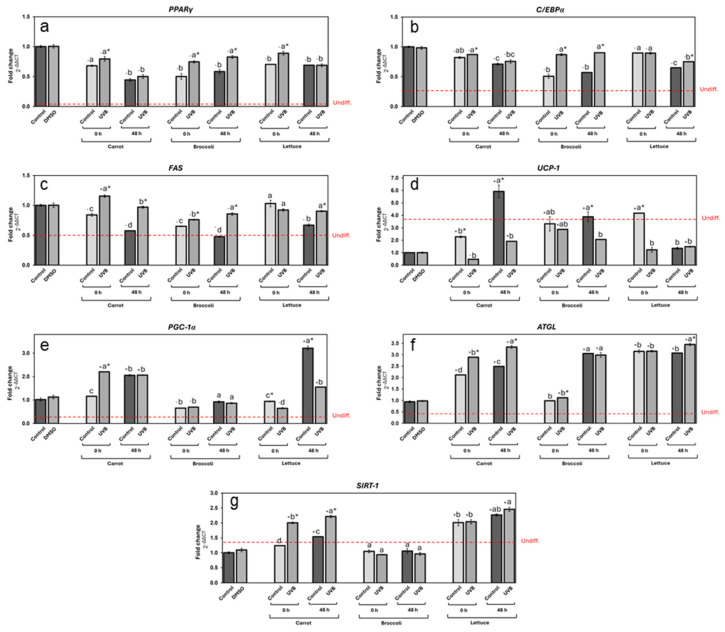
Expression of different genes related to lipid metabolism in differentiated adipocytes treated with carrot, broccoli, and lettuce treated with UVB extracts at 125 µg/mL concentration. Expression of genes PPARγ (**a**), C/EBPα (**b**), FAS (**c**), UCP-1 (**d**), PGC-1α (**e**), ATGL (**f**), and SIRT-1 (**g**) measured for carrot, broccoli, and lettuce extracts for non-treated and UVB-exposed samples. DMSO was used as control. Undifferentiated control gene expression level is indicated by the red line. Data represent the mean of 3 repetitions ± the standard error of the mean. +/− indicates a significant difference of each treatment against DMSO. Different letters among bars (a–d) indicate a statistical difference between treatments within the same vegetable. Asterisk (*) indicates statistical differences between treatments for the same vegetable at the same evaluated time. All statistical differences were defined using the LSD test (*p* < 0.05).

The effects of vegetable extracts on gene expression related to adipogenesis and lipid metabolism were also noteworthy (Figure 6). PPARγ (Figure 6a) and C/EBPα (Figure 6b), two key genes involved in adipocyte differentiation, were significantly upregulated in all treatments compared to undifferentiated cells. Carrot extracts led to a reduction in both PPARγ and C/EBPα expression compared to controls, with stored extracts showing a greater reduction (53% and 27% on average, respectively, contrasting with the vehicle group). The UVB exposure slightly increased the expression of both genes compared to non-UVB-treated extracts. Broccoli and lettuce followed similar trends. Both broccoli controls displayed the most significative gene downregulation (46%, contrasting with the vehicle group). In the case of lettuce extracts, the control 0 h and the storage groups significantly reduced the gene expression of PPARγ, and in the case of C/EBPα expression, only the storage groups downregulated it. In both vegetable cases, the UVB treatment promoted higher expression levels of these genes.

For the FAS gene (Figure 6c), which is linked to lipogenesis, the expression was generally reduced by vegetable extracts compared to the vehicle control, in a similar fashion as PPARγ and C/EBPα. Interestingly, all three stored vegetable control extracts showed a higher reduction in gene expression, highlighting the stored broccoli control extract as the most efficient, reducing by 52% the FAS expression in contrast with the vehicle group. In the same way, the UVB-treated extracts showed higher expression than non-UVB-treated ones, particularly in the case of carrots at 0 h, where UVB treatment led to a significant increase in FAS expression.

Thermogenesis-related genes, such as UCP-1 (Figure 6d) and PGC-1α (Figure 6e), were also influenced by vegetable extracts. UCP-1 expression was enhanced by vegetable extracts in most cases, particularly after storage (except lettuce). The stored carrot control extract was the most effective upregulating in 555% the UCP1 expression, followed by 318% of the 0 h lettuce extract and, finally, 289% of the stored broccoli extract.

PGC-1α expression (Figure 6e), on the other hand, was increased by UVB treatment in carrots immediately after exposure by 120%, remaining with a similar upregulation after storage. All broccoli extracts had an unnoticeable effect on PGC-1α expression contrasting with the vehicle group. In lettuce extracts, the stored control upregulated the gene expression by 221%, while the UVB treatment led to a reduction in expression at both 0 and 48 h, although a significant increase was observed after storage.

Finally, the lipolysis-related genes ATGL (Figure 6f) and SIRT-1 (Figure 6g) were significantly impacted by vegetable extracts, with UVB exposure leading to notable increases in expression in carrots, particularly after storage, which upregulated its expression by 224% and 121%, respectively, compared with the vehicle group. The extracts from stored broccoli only showed a significant increase in ATGL expression (202%, on average). All lettuce extracts displayed an increase in ATGL and SIRT-1 expression (220% and 119% on average, respectively, compared with the vehicle group). No significant changes were observed between the UVB-treated extract and control at 0 h; however, significant ATGL and Sirt1 upregulation was observed at 48 h. Interestingly, ATGL and Sirt1 expression followed similar patterns, enhancing their expression following UVB exposure and storage in most cases, except for the broccoli extracts.

These findings are also in agreement with studies previously published [41,46]. The modulation of gene expression related to adipogenesis and lipid metabolism in 3T3-L1 cells provides insights into the mechanisms by which vegetable extracts influence adipocyte function. Similarly to the previous section, a probable hormesis phenomenon of phenolic compounds, including CHA, influenced gene regulation after the UVB treatment [53]. For instance, PPARγ and C/EBPα, two critical transcription factors in adipogenesis, as well as FAS, a key enzyme in adipose tissue triglyceride accumulation, were upregulated in response to UVB-treated extracts and, to a lesser extent, in the stored extracts, indicating a complex interplay between these phenolic compounds and the cellular pathways regulating fat cell differentiation [56].

This differential response of UVB-treated extracts highlights the complexity of how UVB radiation influences the phenolic compounds and other bioactive molecule synthesis in vegetables [17]. It suggests that UVB radiation may enhance or modify the extract’s ability to influence adipogenic pathways, potentially by altering the chemical composition or availability of specific active compounds.

The significant upregulation of ATGL and SIRT-1 in response to storage and UVB treatment, particularly in lettuce and carrots, adds further weight to the potential of these extracts to regulate lipid catabolism [57]. Given that SIRT-1 is involved in mitochondrial function and metabolic regulation, its upregulation alongside ATGL suggests that vegetable-derived phenolic compounds could improve lipid breakdown and mitochondrial efficiency, providing a novel strategy for managing metabolic conditions such as obesity and its associated comorbidities [58].

Altogether, these data indicate that UVB radiation may shift the balance between fat cell differentiation and lipid accumulation, promoting adipogenesis while simultaneously reducing lipid deposition. This dual effect could be harnessed therapeutically to limit excessive lipid storage while maintaining necessary fat cell functions. By strategically leveraging the hormetic effects of these compounds, it may be possible to develop interventions that promote beneficial metabolic adaptations, including reduced fat accumulation, enhanced lipid metabolism, and increased thermogenesis. The study suggests that UVB radiation enhances the bioactivity of vegetable extracts, particularly fat breakdown promotion. Further research is needed to fully understand the mechanisms by which UVB radiation alters the bioactivity of phenolic compounds and to determine the optimal conditions for harnessing these effects in therapeutic interventions [55]. 

## 4. Conclusions

The findings of this study demonstrate that UVB radiation is an effective tool for significantly enhancing the bioactivity of vegetables, improving their content of bioactive compounds such as phenolics and chlorogenic acid. The developed at-home UVB device proved to be successful in increasing the nutraceutical content of common vegetables like carrots, lettuce, and broccoli, with optimized conditions leading to substantial increases in antioxidant, anti-inflammatory, and anti-obesogenic properties. Additionally, the device effectively stimulated key gene expressions related to lipid metabolism, such as UCP-1, SIRT-1, and ATGL, further enhancing the health benefits of UVB-treated vegetables. This approach offers a practical and convenient method for consumers to boost the nutritional quality of their vegetables, presenting a novel strategy for addressing diet-related health issues through enhanced bioactive compound intake.

## 5. Patents

Patent (MX/a/2024/010935) and industrial design (MX/f/2024/002692) applications have been made to the Mexican Institute of Industrial Property (IMPI, by its acronym in Spanish).

## Figures and Tables

**Table 1:** Model coefficients for total phenolic content in carrot, broccoli, and lettuce as affected by UVB exposure. Third-order polynomial regression model coefficients are shown for treated vegetables after UVB exposure (0 h) and after storage at 15 °C (48 h). The coefficient of determination is indicated for each model.

	Carrot		Broccoli		Lettuce	
	0 h	48 h	0 h	48 h	0 h	48 h
*β*_0_ ^1^	42.56	197.28	140.56	118.33	16.39	11.80
*β*_1_ ^1^	6.24	−38.40	−1.93	0.98	−0.63	0.94
*β*_2_ ^1^	3.76	−32.68	−8.57	5.42	0.54	0.67
*β*_3_ ^1^	−0.36	3.32	0.18	−0.14	0.07	−0.07
*β*_4_ ^1^	−0.85	5.97	1.83	−1.28	−0.17	−0.12
*β*_5_ ^1^	0.34	7.12	0.65	−0.01	0.06	−0.53
*β*_6_ ^1^	0.01	−0.07	−0.01	0.01	−0.01	0.01
*β*_7_ ^1^	0.05	−0.28	−0.10	0.07	0.01	0.01
*β*_8_ ^1^	−0.01	−0.21	−0.01	0.01	−0.01	0.02
*β*_9_ ^1^	−0.03	−0.52	−0.06	0.01	−0.01	0.05
*β*_10_ ^1^	0.01	0.02	0.01	−0.01	0.01	−0.01
R^2^	0.97	0.79	0.89	0.79	0.77	0.93

^1^ *β*_0_, intercept; *β*_1_, linear term for time (min); *β*_2_, linear term for intensity (W/m^2^), *β*_3_, quadratic term time (min); *β*_4_, quadratic term for intensity (W/m^2^), time (min); *β*_5_, linear interaction term between time and intensity; *β*_6_, cubic term for time; *β*_7_, cubic term for intensity; *β*_8_, for quadratic–cubic interaction term for time–intensity; *β*_9_, for quadratic–cubic interaction term for intensity–time; *β*_10_ for quadratic–quadratic interaction term.

## Data Availability

The original contributions presented in the study are included in the article/Supplementary Materials, further inquiries can be directed to the corresponding author.

## Supplementary Materials

The following supporting information can be downloaded at: https://www.mdpi.com/article/10.3390/foods13203311/s1, Figure S1: Cytotoxicity assays for carrot, broccoli, and lettuce extracts on Caco-2 (a), Raw 264.7 (b), and 3T3-L1 (c) cells; Table S1: Primers to be used for the quantification of gene expression in differentiated 3T3-L1 cells exposed to the vegetable extracts.
