# Supplementary material for: Boosting Health Benefits in Vegetables: A Novel Ultraviolet B (UVB) Device for Rapid At-Home Enhancement of Phytochemicals and Bioactivity
Source: Foods. 2024 Oct 18;13(20):3311. doi: 10.3390/foods13203311 (PMC11507927; doi:10.3390/foods13203311)
Supplement: Supplementary file 1 [file foods-13-03311-s001.zip › foods-3257180-supplementary.pdf]

foods

MDPI

Supplementary Material

Boosting Health Benefits in Vegetables: A Novel Ultraviolet B (UVB) Device for Rapid At-Home Enhancement of Phytochemicals and Bioactivity

Alejandro Gastélum-Estrada [1], Edwin E. Reza-Zaldivar [2] and Daniel Alberto Jacobo-Velázquez [2,*]

[1] Tecnológico de Monterrey, Escuela de Ingeniería y Ciencias, Campus Guadalajara, Av. General Ramón Corona 2514, Zapopan 45201, Jalisco, México.
* Correspondence: djacobov@tec.mx

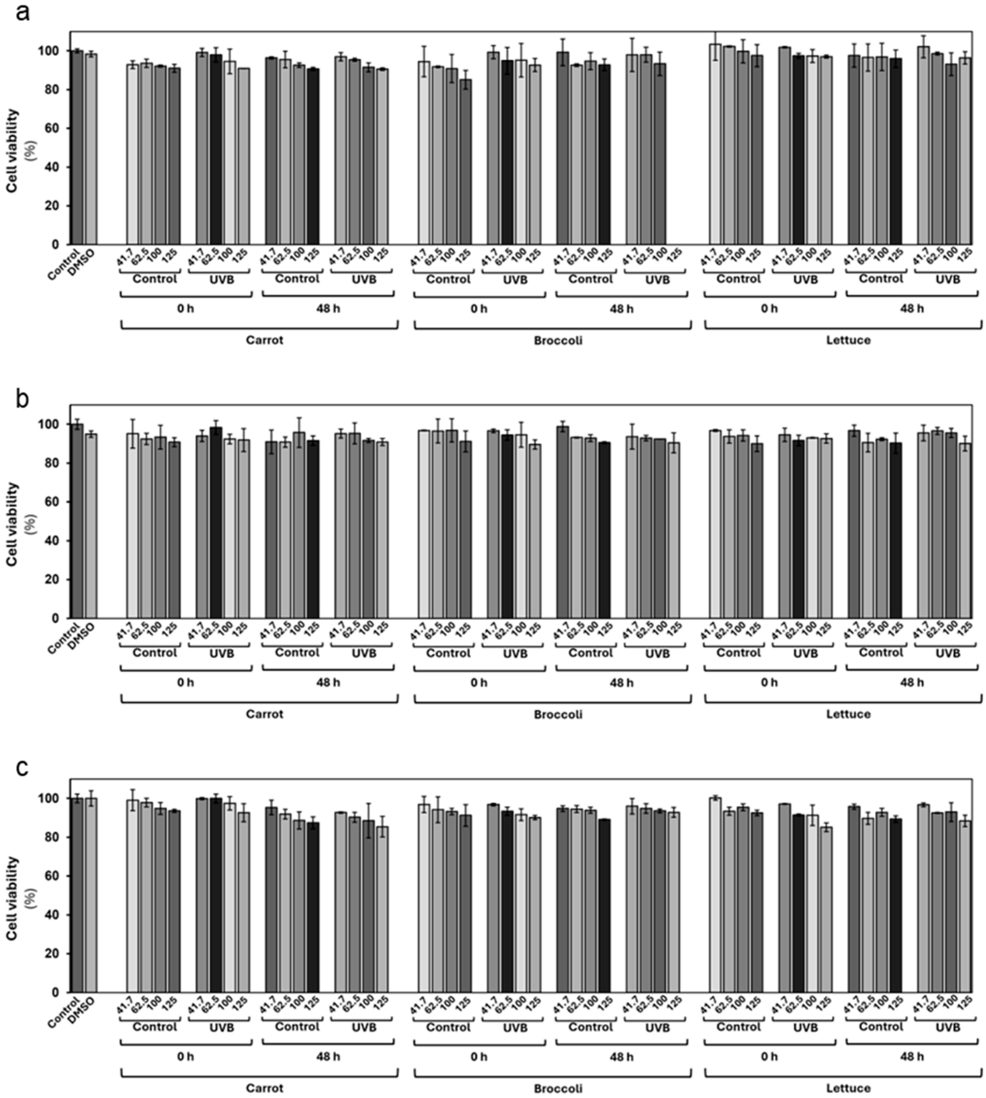

Figure S1. Cytotoxicity assays for carrot, broccoli, and lettuce extracts on Caco-2 (a), Raw 264.7 (b), and 3T3-L1 (c) cells. DMSO-resuspended extracts at 41.7, 62.5, 100, and 125 µg/mL concentrations were evaluated for each vegetable immediately after UVB exposure and after 48 h storage at 15°C. Data represents the mean of 3 repetitions ± the standard error of the mean.

Table S1. Primers used for the quantification of gene expression in differentiated 3T3-L1 cells exposed to the vegetable extracts.

Gene	Primer	Length
PPARγ	F: CAAGAATACCAAAGTGCGATCAA	23
	R: GAGCTGGGTCTTTTCAGAATAATAAG	26
C/EBPα	F: CTGGAAAGAAGGCCACCTC	19
	R: AAGAGAAGGAAGCGGTCCA	19
FAS	F: GATCCTGGAACGAGAACAC	19
	R: AGACTGTGGAACACGGTGGT	20
UCP-1	F: ACGGGGACCTACAATGCTTAC	21
	R: CAGCTTGGTACGCTTGGGTAC	21
PGC1α	F: TGTTCCCGATCACCATATTCC	21
	R: AGCTGTCGTACCTGGGCCTAC	21
ATGL	F: TTCACCATCCGCTTGTTGGAG	21
	R: AGATGGTCACCCAATTTCCTC	21
SIRT-1	F: GACCTCCCAGACCCTCAAGC	20
	R: TGTGACACAGAGACGGCTGG	20
B-ACTIN	F: AGGCCAACCGTGAAAAGATG	20
	R: TGGCGTGAGGGAGAGCATAG	20
